# The Polish Version of the Parental Assistance with Child Emotion Regulation (PACER) Questionnaire: Preliminary Psychometric Properties and Links with Parental Burnout, Mental Health Outcomes, and Emotion Beliefs

**DOI:** 10.3390/children12111428

**Published:** 2025-10-22

**Authors:** Paweł Larionow, Monika Mazur, Natalia Pilarska, Karolina Mudło-Głagolska, Dorota Szczygieł, David A. Preece

**Affiliations:** 1 Institute of Psychology, University College of Professional Education, 53-329 Wrocław, Poland; karolina.mudlo-glagolska@wskz.pl; 2 School of Human Sciences, VIZJA University, 01-043 Warsaw, Poland; monique.mazur@gmail.com; 3 Faculty of Psychology, Kazimierz Wielki University, 85-064 Bydgoszcz, Poland; pilarska@ukw.edu.pl; 4 Faculty in Sopot, SWPS University, 81-745 Sopot, Poland; dszczygiel@swps.edu.pl; 5 School of Population Health, Curtin University, Perth, WA 6102, Australia; david.preece@curtin.edu.au; 6 Department of Psychology, Stanford University, Stanford, CA 94305, USA; 7 School of Psychological Science, The University of Western Australia, Perth, WA 6009, Australia

**Keywords:** anxiety, child emotion regulation, depression, emotion beliefs, parental burnout, psychometric properties, psychopathology, questionnaire, somatic complaints, well-being

## Abstract

**Background/Objectives**: This study presents a brief report on the preliminary psychometric properties of a first Polish version of the Parental Assistance with Child Emotion Regulation (PACER) Questionnaire. The PACER measures ten emotion regulation (ER) strategies parents use to assist their children in their ER. We aimed to examine PACER’s internal consistency reliability, convergent, divergent and discriminant validity. **Methods**: The sample included 74 Polish-speaking parents aged from 27 to 50, recruited in 2025. Along with the PACER, we used a robust set of psychometric tools for measuring parental burnout, anxiety and depression symptoms, somatic complaints, well-being, and beliefs about emotions. **Results**: All PACER subscale scores demonstrated good-to-excellent internal consistency reliability (i.e., Cronbach’s alpha of ≥0.83). Encouraging adaptive strategies (e.g., reappraisal) in one’s children was associated with better outcomes (e.g., lower parental burnout and psychopathology symptoms), whereas maladaptive strategies (e.g., avoidance) were associated with worse outcomes. We also demonstrated that PACER strategy scores were statistically separable from maladaptive beliefs about emotions, indicating good discriminant validity. **Conclusions**: Overall, the Polish PACER demonstrated promising psychometric properties and strong clinical relevance. These findings can help to inform interventions targeted at improving parents’ capacity to help their children regulate emotions, which in turn may help to prevent parental burnout.

## 1. Introduction

Emotion regulation is a multifaceted construct referring to the strategies people use to manage the onset, intensity, duration, and expression of emotional responses [1,2]. Conceptualized as a dynamic, multicomponent process, emotion regulation enables individuals to influence which emotions they experience, when they arise, and how they are expressed [3]. In childhood, emotion regulation is crucial for socio-emotional development and represents a key predictor of lifelong mental health and well-being [4,5]. Research shows that effective emotion regulation can serve as a protective factor, shielding children from psychopathologies, such as depression or anxiety disorders, when they face adverse conditions [6,7,8]. Conversely, early difficulties in emotion regulation are associated with internalizing and externalizing problems in adolescence, suggesting emotion regulation as a developmental pathway from early vulnerability to later maladjustment [9,10]. Collectively, these findings indicate that while difficulties in emotion regulation heighten vulnerability to psychopathology, effective emotion regulation promotes resilience and supports children’s social, academic, and personal thriving [11,12].

Parents play a central role in developing children’s emotion regulation. They shape regulatory pathways through emotion-related socialization behaviors, including responses to children’s emotional expressions, their own emotional behavior, and conversations about emotions [13,14]. Furthermore, parents model and guide specific regulation strategies [4]. Parents’ own regulatory skills are crucial; evidence shows that parents’ emotional management abilities affect both their quality of parenting and children’s adjustment [15]. These interactions are reciprocal: children’s emotional expressions can influence parental reactions, creating processes that guide socio-emotional development [16,17]. Thus, parental assistance with emotion regulation can be seen as a key mechanism for fostering adaptive coping and transmitting regulatory patterns across generations [18,19].

For measuring parental assistance with child emotion regulation, several questionnaires were developed (for review, see Cohodes et al. [19]). In this study, we focus on the *Parental Assistance with Child Emotion Regulation (PACER) Questionnaire*, a 50-item self-report questionnaire aimed at assessing how parents support their children in managing negative emotions (i.e., what emotion regulation strategies they try to help their children to implement) [19]. Based on the *extended process model of emotion regulation* [1,2], the PACER assesses 10 different strategies: behavioral disengagement, problem solving, social support search, rumination, distraction, reappraisal, acceptance, expressive suppression, venting, and avoidance. Across these strategies, it is possible to distinguish between adaptive and maladaptive parental assistance, with some strategies generally associated with better outcomes than others. This comprehensive approach makes the PACER effective in examining parental scaffolding of children’s regulatory abilities [20].

Initial validation studies show significant associations between parental assistance and children’s adjustment. Greater use of strategies generally regarded as adaptive, such as problem solving, social support search, or reappraisal, is linked to fewer internalizing and externalizing symptoms, whereas reliance on strategies generally considered maladaptive, such as expressive suppression or rumination, is associated with poorer socio-emotional outcomes [19]. Moreover, PACER scores correlate with parents’ own emotion regulation tendencies, indicating that caregivers’ regulatory styles may shape how they assist their children. For example, parents who rely more on cognitive reappraisal in managing their own emotions are more likely to encourage this strategy in their children, whereas parents who habitually use expressive suppression are more likely to scaffold this form of regulation as well. Notably, higher parental stress and internalizing symptoms are associated with greater reliance on less effective strategies for alleviating negative affect, as captured by the PACER [19].

Despite its potential, the PACER has not yet been validated in Polish-speaking populations. This represents an important gap given cultural differences in emotion socialization practices [21] and the growing interest in parental factors linked to child adjustment in Poland [22,23]. As such, establishing the psychometric properties of different language versions of the PACER, such as in Polish, is both timely and important. Emotion regulation is one of the most important emotion-based constructs, theoretically associated with a lot of other emotional constructs (e.g., beliefs about emotions) or psychological outcomes [24]. As such, there is a need to further explore links between the PACER and parental mental health outcomes, such as parental burnout, anxiety and depression symptoms, somatic complaints, and well-being, as well as parents’ beliefs about emotions. Based on the Ford and Gross’s framework [24], theorizing that maladaptive beliefs about emotions can lead to worse psychological outcomes through impairments in emotion regulation processes, we found the examination of these links theoretically justified and practically useful. Such relationships may provide clinically relevant insights into the interplay between parents’ well-being and the ways in which they guide their children’s emotional regulation.

The present study therefore aimed to conduct a preliminary examination of the psychometric properties of the PACER in a Polish sample. Specifically, we assessed its internal consistency and latent structure, and we explored associations with parents’ parental burnout, mental and somatic health indicators, well-being, as well as beliefs about emotions in a sample of Polish parents.

We expected that parents’ psychological functioning would be associated with the ways in which they assist their children in regulating negative emotions. As such, based on the theory and the above-described research, we have formulated the following hypotheses:

**Hypothesis** **1.** *All ten PACER subscales would exhibit good internal consistency reliability.*

**Hypothesis** **2.** *The PACER would demonstrate good convergent and divergent validity, with the more frequent use of adaptive strategies being associated with better parental outcomes (*i.e.*, lower levels of parental burnout, lower levels of psychopathology symptoms, somatic complaints, and maladaptive emotion beliefs, as well as higher levels of well-being), and with the more frequent use of maladaptive strategies being related to worse outcomes.*

**Hypothesis** **3.** *The PACER would demonstrate strong discriminant validity (*i.e.*, statistical separability) against the maladaptive emotion beliefs construct.*

As for hypothesis-generating and agenda-setting analyses, we were interested in investigating latent structure of ten PACER strategies. That is, we examined whether these strategies would group into broader categories like adaptive and maladaptive strategies, thus indicating the potential for calculating higher-order composite scores. In terms of other exploratory analyses, we investigated demographic differences, analyzing links of PACER strategies with parents’ age and education levels.

## 2. Materials and Methods

### 2.1. Procedure

This research was conducted in accordance with the Declaration of Helsinki Ethical Principles. The study was approved by the Ethics Committee of the Faculty of Psychology of the Kazimierz Wielki University (reference 1/13 June 2022 and its later revision with a reference 4/10 June 2025). Collected data were secured according to the requirements of the above ethics committee.

Our participants were recruited mainly at Polish universities across social science students in June and July 2025. They were informed about this online anonymous survey during outside class activities (e.g., in the time of workshops not related to parenting). Our respondents obtained relevant pieces of information regarding this study, including a criterion to participate (i.e., being a parent of a child until the age of 18), and consequently received a link with the survey by a Google Forms platform with an appended consent form, and were asked to fill it out. All participants provided their written informed consent digitally before completing the survey. No reimbursement for participation was provided.

Our pre-defined inclusion criteria were: (1) Polish-speaking people aged 18 years or over, who signed their informed consent for study participation, (2) being a parent of a child or children until the age of 18, and (3) spending at least 1 h per day in direct contact with their child(ren). Our pre-defined exclusion criteria were: (1) inattentive responding (based on the below-described attention check questions), and (2) missing or incorrect data in a sociodemographic form (all replies in the survey were mandatory, missing data on questionnaire items were not possible). This was a pilot study; therefore, two reasonable minor exceptions to individual inclusion criteria were permitted. Hence, we decided to include a parent who had their youngest child aged 22 but who spent a significant amount of time (i.e., at least 1 h per day in direct contact with their children) to express parental assistance, as well as another parent, who did not indicate their age (i.e., missing data).

We used a series of measures to control the data quality. Four attention check questions were used. Three of them were a request to indicate a specific reply in a query (“In this attention check question, please choose a reply of strongly agree”). The requested replies (e.g., *strongly agree* or *disagree*) were different in each of the three questions. We posted the fourth attention check question (“Please indicate whether you have filled out a survey reliably and attentively”) at the end of the survey. Two replies (i.e., agreement or disagreement) to this question were provided. While all participants agreed that they filled out the survey reliably and attentively, not all of them replied to the previous attention check questions correctly. As such, a total of 84 people completed the survey, however, 9 participants, who failed to reply to at least one question correctly, were excluded from the analyses. One individual indicated that they had no child, therefore, they were also excluded. Hence, the final sample comprised 74 parents. To check the data quality further, we also analyzed a difference between the age of a parent and the age of their oldest/younger child.

### 2.2. Participants

The sample consisted of 74 Polish-speaking parents, including 70 mothers and 4 fathers, with ages ranging from 27 to 50 (*M* = 39.48; *SD* = 5.63), living in the same household with at least one child. The number of children ranged from 1 to 7 (*M* = 2.22, *SD* = 1.13), with majority being parents of 2 children (see Table 1). The average age of the youngest (or only) child was around 8 years (*M* = 7.66, *SD* = 4.97), whereas the average age of the eldest child was around 11 years (*M* = 11.42, *SD* = 5.50). A total of 12 parents (16.22% of the total sample) reported bringing up a child with disability. The studied parents reported spending in direct contact with their child(ren) on average 6.57 h per day (*SD* = 3.57). Participants’ places of residence were located in 14 (out of 16) voivodeships of Poland. Detailed sociodemographic and parental characteristics of the sample are demonstrated in Table 1.

### 2.3. Measures

We used a demographic questionnaire to collect data on basic demographic characteristics (e.g., age, gender, etc.) and features related to parenting (e.g., a family type, a number of children, etc.). A series of validated measures in their Polish versions was used to assess psychological constructs as correlates of parental assistance with child emotion regulation. Questionnaire descriptions were provided below, whereas their internal consistency reliability in this study was indicated in Table 2.

#### 2.3.1. The Parental Assistance with Child Emotion Regulation (PACER) Questionnaire and Its Translation

The PACER is a 50-item self-report questionnaire for measuring ten emotion regulation strategies used by parents to assist emotion regulation of their children [19]. The PACER consists of ten subscales, each containing five items. Across ten PACER strategies are: (1) Behavioral disengagement (e.g., “I remove my child from a situation when it is causing them to have negative feelings”), (2) Problem solving (e.g., “I help my child think carefully about different solutions to their problems”), (3) Social support search (e.g., “I help my child find other people to help them (including myself)”), (4) Rumination (e.g., “I help my child replay whatever is making them have negative feelings in their mind”), (5) Distraction (e.g., “I help my child find ways to distract themselves from their negative feelings”), (6) Reappraisal (e.g., “I help my child try to see the positive aspects of a situation that is making them have negative feelings”), (7) Acceptance (e.g., “I help my child understand that it’s okay to have negative feelings”), (8) Expressive suppression (e.g., “I help my child to not show their negative feelings”), (9) Venting (e.g., “I help my child talk about the situation or problem that caused them to feel this way”), and (10) Avoidance (e.g., “I help my child stay away from entering situations that might make them have negative feelings”). Items are scored on a 7-point scale from 1 (*strongly disagree*) to 7 (*strongly agree*). Subscale scores range from 5 to 35, with higher scores indicating the higher strategy use [19].

Prior to the use of the PACER in a Polish context, we obtained a permission from the creators of the original PACER [19]. To develop the Polish version of the PACER, we followed standard and well-recognized translation procedures [25]. First, the English version of the PACER was translated into Polish by three independent translators. Then, these translations were merged into a single Polish version. Afterwards, an independent translator conducted its back translation into English. This translation was sent to the developers of the English PACER to collate it with the original questionnaire. One of the PACER’s creators, Dylan Gee [19], reviewed this back translation, and suggested minor edits. We carefully analysed this feedback, and made appropriate minor adjustments to the questionnaire, resulting in the pre-final Polish version of the PACER. Next, this pre-final version was distributed to ten Polish-speaking parents (both mothers and fathers) with different demographic and parenting backgrounds to collect their feedback on the clarity and comprehensibility of this prefinal Polish version of questionnaire. After collecting their critique, we made very minor changes to the pre-final version, resulting in the final Polish version. This final authorised questionnaire was administered in this study, and is provided in the Supplementary Materials.

#### 2.3.2. The Parental Burnout Assessment (PBA)

The PBA is a 23-item self-report questionnaire for measuring parental burnout [26]. The PBA consists of four subscales: (1) exhaustion in parental role (e.g., “I have the sense that I’m really worn out as a parent”), (2) emotional distancing (e.g., “I do what I’m supposed to do for my child(ren), but nothing more”, (3) feelings of being fed up (e.g., “I can’t stand my role as father/mother any more”), and (4) contrast in parental self (e.g., “I tell myself that I’m no longer the parent I used to be”). While subscale scores can be calculated, computing a total PBA score, representing an overall marker of parental burnout, is more frequently assessed in research. Hence, in this study, we calculated the total PBA score only. Items are scored on a 7-point scale from 0 (*never*) to 6 (*every day*). The total score ranges from 0 to 138, with higher scores indicating a higher level of parental burnout [26]. The PBA has empirically-derived cut-off scores of different levels of parental burnout. PBA total scores from 52.7 to 86.3 serve as indicators of moderate parental burnout, whereas scores of >86.3 indicate severe parental burnout [27]. In this study, the Polish version of the PBA was used [28].

#### 2.3.3. The Patient Health Questionnaire-4 (PHQ-4)

The PHQ-4 is a 4-item self-report questionnaire for measuring anxiety and depression symptoms over the previous two weeks [29,30]. The PHQ-4 consists of two subscales: anxiety (2 items; e.g., “Not being able to stop or control worrying”) and depression (2 items; e.g., “Little interest or pleasure in doing things”). Along with these subscale scores, a total sum PHQ-4 score is calculated as an overall marker of psychological distress. Items are scored on a 4-point scale from 0 (*not at all*) to 3 (*nearly every day*). Subscale scores range from 0 to 6, whereas a total score ranges from 0 to 12, with higher scores indicating higher levels of measured symptoms [29,30]. In this study, the Polish version of the PHQ-4 was used [31].

#### 2.3.4. The Giessen Subjective Complaints List-8 (GBB-8)

The GBB-8 is an 8-item self-report questionnaire for measuring the eight most frequently assessed somatic complaints (e.g., “Being easily exhausted”; “Tiredness”; “Feeling bloated or distended”, or “Stomachache”) [32,33]. Four 2-item subscale GBB-8 scores, representing four categories of somatic complaints, as well as a total GBB-8 score, representing general somatic symptom burden, can be computed. Given that the somatic complaints construct was not central in this study, and individual subscale scores demonstrate somewhat lower internal reliability in research [34], we calculated only the total score in this study. Items are scored on a 4-point scale from 0 (*not at all*) to 4 (*very much*). The total score ranges from 0 to 32, with higher scores indicating a higher level of somatic complaints [32,33]. In this study, the Polish version of the GBB-8 was used [34].

#### 2.3.5. The WHO-Five Well-Being Index (WHO-5)

The WHO-5 is a 5-item self-report questionnaire for measuring positive well-being over the previous two weeks [35]. Items (e.g., “I have felt calm and relaxed”) are scored on a 6-point scale ranging from 0 (*at no time*) to 5 (*all the time*). A total sum WHO-5 score, ranging from 0 to 25, is computed, with higher scores indicating a higher level of well-being [35]. In this study, the Polish version of the WHO-5 was used [36,37].

#### 2.3.6. The Emotion Beliefs Questionnaire (EBQ)

The EBQ is a 16-item self-report questionnaire for measuring maladaptive beliefs about emotions [38]. The EBQ assesses beliefs about the uncontrollability and uselessness of negative and positive emotions separately. The EBQ consists of four subscales: (1) Negative-Controllability (e.g., “Once people are experiencing negative emotions, there is nothing they can do about modifying them”), (2) Positive-Controllability (e.g., “People cannot control their positive emotions”), (3) Negative-Usefulness (e.g., “There is very little use for negative emotions”), and (4) Positive-Usefulness (e.g., “Positive emotions are very unhelpful to people”), as well as several composite scores, including a total scale score. In this study, we calculated subscale scores only, as we wanted to explore how specific emotion beliefs across positive and negative emotions are associated with the PACER strategies. Items are scored on a 7-point scale, ranging from 1 (*strongly disagree*) to 7 (*strongly agree*). Subscale scores range from 4 to 28, with higher scores indicating stronger beliefs that emotions are uncontrollable and useless [38]. In this study, the Polish version of the EBQ was used [39].

### 2.4. Analytic Strategy

Statistical analyses were carried out using *LibreOffice* 25.2 for data preparation and *JASP* 0.95 [40] for main analyses. Descriptive statistics, including skewness and kurtosis as well as min. and max. values, were calculated to assess the distribution characteristics (e.g., variability) [41] of individual PACER subscales.

To estimate internal consistency reliability, McDonald’s omega and Cronbach’s alpha coefficients with 95% confidence intervals (95% CIs) were computed. For these reliability coefficients, values ≥ 0.70 were treated as acceptable, ≥0.80 as good, and ≥0.90 as excellent [42].

To assess convergent and divergent validity, Pearson correlations between PACER scores and other psychological constructs (e.g., parental burnout) were calculated. Overall, we predicted that the more frequent use of adaptive PACER strategies (e.g., problem solving, social support search) would be related to more favorable parenting and mental health outcomes (e.g., lower levels of parental burnout, psychopathology symptoms). Conversely, we expected that the more frequent use of maladaptive PACER strategies (e.g., avoidance, rumination) would be related to less favorable parenting and mental health outcomes. As we had preplanned hypotheses with planned correlations, we did not apply any correction for multiple correlations [43].

We were also interested in examining the latent structure of the PACER. That is, we aimed to explore whether ten PACER strategies grouped into broader categories or composite scores, like adaptive or maladaptive strategies. To do so, we conducted a second-order exploratory factor analysis [44] with all ten PACER subscale scores. According to a traditional rule of thumb in exploratory factor analysis, a ratio of 5 participants per variable is used [45]. As such, our sample (*n* = 74) was enough to conduct an analysis with 10 variables. Factors were selected on the basis of parallel analysis (established on the correlation matrix and factor eigenvalues), with the principal axis factoring method and varimax rotation. To assess suitability of our data for factor analysis, we used the Kaiser–Meyer–Olkin (KMO) test and Bartlett’s test, with KMO values of >0.50 and a significant Bartlett’s test (at least *p* < 0.05) indicating appropriateness of the data for factor analysis [46].

Additionally, we strove to examine the discriminant validity of the PACER against emotion beliefs. In other words, we aimed at examining the statistical separability of the parental assistance with emotion regulation construct from beliefs about emotions. As such, we applied the same analysis as presented above, but conducting it with all ten PACER subscale scores and four EBQ subscale scores. According to the above-mentioned rule of thumb, our sample was sufficient to conduct this exploratory factor analysis with 14 variables. We expected that the PACER subscale scores would not load on the same factor(s) composed of emotion beliefs subscales. For completeness and comparability reasons, we conducted the same set of exploratory factor analyses with the oblimin rotation [47], assuming factors were correlated. Nonetheless, changing rotation from varimax to oblimin did not change the results substantially; therefore, for parsimoniousness reasons, we decided to demonstrate the results with the varimax rotation only.

We were also interested in examining demographic differences in parental assistance with child emotion regulation. For this purpose, we calculated Pearson correlations between parents’ age and PACER scores. We used multivariate analysis of variance to compare the use of ten PACER subscale scores between parents with secondary education and parents with higher education.

## 3. Results

### 3.1. Descriptive Statistics

Table 2 demonstrates descriptive statistics of all the study variables in the total sample (*n* = 74). In these data, the most frequently used PACER strategy was acceptance (*M* = 31.47), followed by problem solving (*M* = 30.66), and social support search (*M* = 30.54), with these strategies showing the least variability of their min. and max. scores. The least frequently used strategy was expressive suppression (*M* = 10.72), followed by rumination (*M* = 11.99), with these two strategies showing a full variability across the range of potential subscale scores.

In terms of understanding the current sample’s specificity, we used the two above-described PBA cut-off scores (see Section 2.3.2) to determine the moderate and severe levels of parental burnout in these data. A total of 8 parents (10.81% of the total sample) had moderate levels of parental burnout levels, whereas 6 parents (8.11%) had severe levels of burnout. As such, 18.92% of the examined parents had clinically significant levels of parental burnout. While these preliminary descriptive analyses should be interpreted with caution, these specific descriptions of the examined sample are informative and may be used for cross-cultural comparative analyses.

### 3.2. Internal Consistency Reliability

Internal consistency reliability of all PACER subscales was good to excellent, with McDonald’s omega of ≥0.83 and Cronbach’s alpha of ≥0.83. The other questionnaires demonstrated acceptable to excellent reliability, with omega and alpha coefficients of ≥0.70, except the PHQ-4 Anxiety scores and EBQ Negative-Controllability scores, with somewhat lower but still acceptable reliability coefficients of 0.61 and 0.67, respectively.

### 3.3. Convergent and Divergent Validity

As expected, PACER subscale scores were differentially associated with parental burnout, psychopathology and somatic symptoms, well-being as well as maladaptive emotion beliefs (see Table 3). Venting, reappraisal, and social support search acted as adaptive strategies, with 7 to 5 significant links with correlates used. In contrast, behavioral disengagement and distraction (each with 1 link with correlates) as well as rumination (2 links with correlates) were associated only with individual maladaptive emotion beliefs. In terms of parental burnout, the higher usage of reappraisal, social support search and problem solving were associated with lower levels of parental burnout, whereas the higher usage of avoidance was associated with higher levels of parental burnout. Detailed results are demonstrated in Table 3.

Overall, our results support good convergent and divergent validity of the Polish PACER. Two PACER composite scores (i.e., adaptive and maladaptive strategies, see the below section for details about the factor analytic basis for these composites) were reasonably associated with individual correlates, supporting their use in research (see Cave-Freeman et al. [48]). The correlation between the composite scores of adaptive strategies and maladaptive strategies was negligible (*r* = 0.02, *p* > 0.05), suggesting these strategies were orthogonal in these data.

### 3.4. Latent Structure of Ten PACER Strategies and Discriminant Validity Against Emotion Beliefs

We examined a latent structure of the PACER, with ten PACER subscales input to our exploratory factor analysis. The KMO test (overall KMO = 0.76) and Bartlett’s test (*Χ*^2^(45) = 267.62, *p* < 0.01) indicated suitability of these data for factor analysis. The exploratory factor analysis of ten PACER subscales extracted two meaningful factors: Factor 1, representing adaptive strategies (i.e., social support search, reappraisal, problem solving, venting, and acceptance), and Factor 2, representing maladaptive strategies (avoidance, expressive suppression, behavioral disengagement, distraction, rumination; see Table 4). For Factors 1 and 2, all factor loadings were high (≥0.62), except for rumination (0.36). There were no cross-loadings. This 2-factor solution explained 48.97% of the total variance. As for the rumination subscale, it demonstrated a somewhat lower factor loading (0.36), but this subscale was still associated with Factor 2 “Maladaptive strategies” and had no cross-loadings on Factor 1 “Adaptive strategies”. The descriptive analysis demonstrated that this was one of the least frequently used strategies across the ten PACER strategies. In our view, this is one of the possible explanations of such a lowered factor loading.

Our results suggest that individual PACER strategies meaningfully group into two broad categories of adaptive and maladaptive strategies, potentially indicating a possibility of calculating two composite PACER scores. These two composite scores (i.e., adaptive and maladaptive strategies) demonstrated excellent internal consistency reliability (see Table 2), further supporting reasonableness of their calculation.

We examined whether the PACER strategies were statistically separable from emotion beliefs. We input ten PACER subscales and four EBQ subscales to our exploratory factor analysis. The KMO test (overall KMO = 0.76) and Bartlett’s test (*Χ*^2^(91) = 457.05, *p* < 0.01) indicated suitability of these data for factor analysis. The exploratory factor analysis of ten PACER subscales and four EBQ subscales extracted three meaningful factors: Factor 1, representing maladaptive strategies, Factor 2, representing adaptive strategies, as well as Factor 3, representing emotion beliefs (see Table 5).

Overall, factor loadings of respective variables in each factor were considerable (≥0.40), except for rumination (0.31). This lowered factor loading of the rumination subscale was discussed in the previous exploratory factor analysis. One significant cross-loading was noted: the EBQ Negative-Usefulness subscale loaded (0.53) on Factor 1 “Maladaptive strategies”, but with the most significant loading (0.62) on its respective Factor 3 “Emotion beliefs”. While this notable cross-loading is present, it may be a statistical artifact in this preliminary analysis rather than potential overlap between the constructs, given the EBQ Negative-Usefulness subscale loaded to a higher extent on its respective Factor 3 “Emotion beliefs”. This 3-factor solution explained 53.66% of the total variance. As such, these results indicate that PACER strategies were statistically separable from people’s beliefs about emotion, supporting good discriminant validity.

### 3.5. Demographic Differences

We preliminarily examined whether age and education were linked to PACER strategies. Age was associated with rumination (*r* = −0.24, *p* < 0.05), whereas other PACER strategies were not related to age (*p* > 0.05). Our multivariate analysis of variance indicated that education levels (secondary versus higher) did not differentiate the use of all PACER strategies (*p* > 0.05). The strong gender imbalance of the sample impeded comparisons between mothers and fathers. Overall, these preliminary demographic associations should be interpreted with caution.

## 4. Discussion

In this pilot study, we demonstrated the preliminary psychometric properties and clinical relevance of the first Polish version of the PACER. Overall, this tool has demonstrated strong psychometric performance, including good-to-excellent internal consistency reliability as well as convergent, divergent, and discriminant validity. Using a series of analyses, we investigated to what extend parents applied different emotion regulation strategies to help their children in regulating emotions, and how the use of such strategies was related to parents’ mental and somatic outcomes. As such, our findings empirically supported good clinical relevance of the PACER.

### 4.1. Distribution Characteristics and Internal Consistency Reliability

The PACER measures ten emotion regulation strategies, which can be used by parents at different frequencies. Based on the descriptive statistics of our data, we assessed to what extent different strategies were used. The most frequently used PACER strategies were acceptance, problem solving, and social support search, which in general emerged as adaptive strategies. In contrast, the least frequently used strategies were expressive suppression and rumination, which were considered maladaptive strategies.

Our results therefore supported previous findings on expressive suppression and rumination as the least frequently used strategies by parents in datasets from the United States of America [19] and Turkey [49], and in the English-speaking sample of caregivers of children aged from 0 to 5 years [20]. Problem solving was the most frequently used strategy in the English [19] and Turkish [49] versions of the PACER, and one of the most used strategies in our Polish sample, and the sample of caregivers [20]. As such, most of response patterns we observed are consistent with past work in other language versions and cultures.

All ten PACER strategy (subscale) scores demonstrated good-to-excellent internal consistency reliability, with McDonald’s omega and Cronbach’s alpha coefficients of ≥0.83. Our results, therefore, supported previous evidence on other language versions of the PACER [19,20,49]. For instance, in the original validation study by Cohodes et al. [19], all PACER subscales had omega and alpha values of ≥0.89. Similarly, the Turkish PACER demonstrated good-to-excellent reliability, with alpha coefficients from 0.81 to 0.94 [49]. As such, our results are in line with other works, suggesting that parental assistance with child emotion regulation, as measured with all ten PACER subscales, can be robustly assessed with this questionnaire in its different language versions.

### 4.2. Links with Parental Burnout, Health, and Emotion Beliefs

In the current study, we investigated how the use of different PACER strategies was associated with a set of other outcome variables, including ones related to parenting (i.e., parental burnout), parents’ health (i.e., psychopathology and somatic symptoms, well-being), and maladaptive emotion beliefs. Overall, we found that PACER strategies usually considered adaptive were indeed related to better parents’ outcomes. Conversely, maladaptive strategies were associated with worse outcomes, thus supporting conclusions from the other studies with the PACER (e.g., [19,49]). As such, parents with higher levels of parental burnout, psychopathology and somatic symptoms, maladaptive emotion beliefs, and lower well-being, were more likely to assist their children with more maladaptive emotion regulation strategies (i.e., behavioral disengagement, rumination, distraction, expressive suppression, and avoidance) and less adaptive ones (i.e., problem solving, social support search, reappraisal, acceptance, venting).

We would like to note that individual PACER subscales demonstrated less or more significant links with other correlates. For instance, behavioral disengagement, distraction, and rumination were the least connected strategies to the correlates used. In contrast, venting, reappraisal, and social support search were the most connected strategies to the correlates. These adaptive strategies seem to be the central strategies across the set of strategies, as measured with the PACER. Overall, the patterns of correlations may suggest the existence of a general trend, with adaptive strategies being the most clinically significant strategies compared to maladaptive strategies. A probable explanation of this trend may be the fact that parents less frequently used maladaptive strategies compared to adaptive strategies in these data. Future studies should examine whether adaptive strategies demonstrate higher clinical relevance over maladaptive strategies.

In sum, PACER strategies seem to be broadly associated with both negative and positive outcomes (i.e., ill-being and well-being). These patterns of correlations support the potential value of targeting parental emotion regulation in clinical settings (e.g., teaching parents to facilitate more adaptive strategies in their children).

### 4.3. Latent Structure, the Use of PACER Composite Scores, and Discriminant Validity

In this study, we examined the latent higher-order structure of the PACER, and based on these analyses, we found preliminary support for calculating two PACER composite scores of adaptive and maladaptive strategies. Previously, Cave-Freeman et al. [48] also provided empirical support for such practice. We justified such plausibility based on the following: (1) two PACER composite scores had excellent internal consistency reliability, (2) our exploratory factor analysis clearly demonstrated that the latent structure of the PACER, at the higher-order level, was composed of two groups of strategies (i.e., adaptive and maladaptive), and (3) the results of convergent and divergent validity with composite scores supported their clinical relevance. Such findings suggest that the use of multiple emotion regulation strategies may be tightly connected (e.g., tending to use clusters of maladaptive or adaptive strategies), coinciding with ideas on polyregulation [50]. However, given the relatively small sample size, the results of our exploratory factor analysis should be considered tentative, not definitive. The observed patterns therefore should be investigated in future studies with larger samples using comprehensive analyses (e.g., a higher-order confirmatory factor analysis).

We noted that PACER strategies were relatively strongly associated with maladaptive emotion beliefs, mainly maladaptive strategies were positively linked with beliefs about the uselessness of negative emotions. Using a second-order factor analysis, we supported that the parental assistance with child emotion regulation construct was statistically separable from parents’ general beliefs about emotions. While these results should be considered preliminary, they have agenda-setting potential. As emotion beliefs are theoretically considered predictors of emotion regulation patterns [51], this finding supports the idea that parents’ maladaptive emotion beliefs may serve as a risk factor for children’s development of psychopathology through the use of less effective parental assistance [19]. Maladaptive emotion beliefs are associated with a wide range of psychopathologies (e.g., [51]), therefore, the examination of this potential mechanism seems to be highly relevant. Potentially, maladaptive emotion beliefs may predict less effective parental assistance with children’s emotion regulation, leading to negative outcomes for both parents and their children. This mechanism is proposed here to be empirically tested in future studies.

### 4.4. Strengths and Limitations of the Study

Whilst this is a brief pilot study, it is considered to have several strengths as well as limitations. The PACER is a novel psychometric tool, with its English [19] and Turkish [49] versions validated so far. As such, this Polish validation study has further supported the high potential of this measure, indicating its cross-cultural comparability.

We used several attention check questions to ensure that our data were of high quality. While the use of such questions has several advantages [52], this may be prone to social desirability bias. To further ensure transparency, we would like to highlight one more time that we applied two reasonable minor exceptions to inclusion criteria, as described in the Section 2.1. Given the pilot nature of this study, we believe these exceptions are reasonable.

To examine PACER’s validity, we used a series of different correlates, like parental burnout or ill-being and well-being health indicators, which were assessed with psychometrically sound measures. However, we did not examine children’s outcomes. In this study, the average age of the youngest (or only) child was around 8 years. While parental assistance with child emotion regulation can vary between children’s developmental stages, due to the pilot nature of the study, we did not implement age-stratified analyses. Future studies should investigate which strategies are less or more frequently used during different developmental stages.

The sample size was relatively small, but still enough for the first examination of basic psychometric properties of the PACER (i.e., internal consistency reliability and convergent and divergent validity). While this sample size was enough for assessing these basic psychometric properties, our exploratory factor analyses may be less conclusive. By their nature, these analyses were preliminary, and we do not treat them as a central part of this study to provide definitive conclusions. We would like to highlight that the required sample size was determined based on the established criteria [45] as described in Section 2.4. While some researchers may treat these criteria [45] as liberal, we believe that they are reasonable for implementing preliminary exploratory factor analysis on a subscale level.

The substantial majority of the participants were mothers, recruited from a social science university. On the one hand, such participants are likely to have more knowledge about efficient emotion regulation, therefore, they can use a more adaptive set of strategies compared to a broader general population of Polish parents. Our participants scored significantly lower on maladaptive emotion beliefs than the normative population of Poles [39]. On the other hand, in our sample, approximately 8% of parents had clinically significant levels of parental burnout. Such a level is common for Polish general parent samples [53], which may indicate that, in terms of burnout, the studied sample was not different from other general samples.

While there is a significant gender imbalance in our sample, we deem this should not be treated as an issue in the assessment of the basic psychometric properties of the PACER (i.e., internal consistency reliability and convergent and divergent validity), which were the central analysis of this paper. It is common practice to assess these properties in the entire sample, without gender differentiation, even in large validation studies. Whilst there is still potential that characteristics of our sample may limit generalizability of our findings to a general Polish population of parents, the patterns of our PACER results are in strong alignment with other cultural populations [19,20,49]. In terms of area of residence, our participants lived in 14 out of 16 voivodeships of Poland, further supporting the characteristics of the study sample as a broad one.

We would like to notice that two subscales of our external measures had relatively low internal consistency reliability, with reliability coefficients of 0.61 and 0.67 for the 2-item PHQ-4 Anxiety subscale and 4-item EBQ Negative-Controllability subscale, respectively. While some psychometricians may treat these values as unacceptable, we believe that for research purposes these reliability coefficients are reasonable, given these subscales represent wide psychological constructs, which are being measured with only two to four items.

It should be noted that several subscales of the Polish PACER had very high internal consistency reliability coefficients (≥0.90), which would have suggested item redundancy (see [54,55]). In our view, PACER subscales measure highly specific psychological constructs (i.e., clearly defined emotion regulation strategies). As such, we do not treat these high coefficients as an item redundancy issue.

We did not test the temporal stability (i.e., test–retest) of PACER scores. Taking into account our promising results on the PACER’s psychometrics, further examination of the questionnaire, including its test–retest reliability, is highly beneficial and assured.

This was a cross-sectional study; therefore, we cannot speculate about cause-and-effect relationships. Speculation about any directionality in relationships is therefore based on theoretical predictions here [1,2,3,24].

### 4.5. Practical Implications

This study has demonstrated the Polish PACER is a psychometrically sound measure, which can be easily applied in a wide range of settings (see Section 4.6). A copy of the Polish questionnaire is provided in the Supplementary Materials, freely available for researchers. Our preliminary assessment of the use of ten PACER strategies helps to provide further insights into how parents’ encouragement of different emotion regulation strategies in their children is an important factor linked to divergent parental outcomes.

Tentatively considering broader implications, the more frequent use of adaptive strategies and less frequent use of maladaptive strategies could be potentially beneficial to decreasing parental burnout, which may be theoretically treated as an outcome of the use of specific PACER strategies. In contrast, based on the theory [24], maladaptive emotion beliefs could be potentially treated as predictors of less adaptive parental assistance with child emotion regulation.

### 4.6. Future Directions

We conducted this pilot study to examine whether the first Polish version of the PACER performed well. We have demonstrated its promising psychometrics; therefore, we believe that collecting more data will be fruitful to further examine the PACER’s factor structure and measurement invariance using confirmatory factor analysis in a large sample of Polish parents, with an equal number of mothers and fathers. Developing an informant form and an observer form of the PACER is highly promising.

As parents may use different PACER strategies to varying extents, identifying different profiles of emotion regulation strategy use via latent profile analysis (see Mancini et al. [20]) seems valuable. Developing norms will also be highly beneficial.

We believe that examination of links between emotion beliefs, parental assistance with child emotion regulation, and parents’ and children’s outcomes is of use for developing psychological interventions aimed at decreasing a risk of psychopathology development for both parents and their children [56]. The PACER can be potentially integrated in such psychological interventions.

Future research should include measures of child’s outcomes to analyze whether parental assistance may explain children’s mental health outcomes (see Cave-Freeman et al. [48]). As beliefs about emotions were reasonably associated with PACER strategies, we deem that a complex assessment of parents’ beliefs about emotions and parental assistance with child emotion regulation is highly beneficial for understanding predictive value of these constructs for children’s and parents’ outcomes. Longitudinal studies are required to examine these mechanisms empirically. Given that parental assistance with child emotion regulation can vary between children’s developmental stages, tracking its stability and changes across these stages is a fruitful research area.

## 5. Conclusions

In this pilot study, the Polish version of the PACER has demonstrated promising psychometric properties, including good-to-excellent reliability of its ten subscales. Convergent and divergent validity was also good, with adaptive and maladaptive emotion regulation strategies being associated as expected with parental ill-being and well-being outcomes. As such, parents who used more adaptive strategies to assist their children in emotion regulation were characterized by lower levels of parental burnout, better mental and somatic health status, as well as lower levels of beliefs that emotion were uncontrollable and useless. In contrast, the more frequent use of maladaptive strategies was conversely associated with these outcomes. Compared to maladaptive strategies, adaptive strategies were more strongly associated with external correlates, potentially demonstrating their higher clinical relevance in these data.

While examining the latent structure of the PACER, we found preliminary support for higher-order composite domains, representing broad groupings of adaptive and maladaptive strategies. Overall, the Polish PACER has exhibited strong psychometric performance, which was comparable to its other language versions. As such, parental assistance with child emotion regulation can be robustly evaluated by the PACER. Given its high cross-cultural comparability, future work on this topic is of high applicability and clinical value. Given the pilot nature of this study, future research in large and diverse samples is required. Cross-cultural examination of the PACER is crucial for consolidating this tool as a globally relevant instrument.

## Figures and Tables

**Table 1:** Demographic characteristics of the study sample.

Demographic Categories		*n*	%
Age (years)	*M* = 39.48; *SD* = 5.63; min. = 27; max. = 50	73	100
Gender	Females	70	94.59
	Males	4	5.41
Area of residence	Large cities (above 100,000 inhabitants)	22	29.73
	Towns (from 20,000 to 100,000 inhabitants)	13	17.57
	Small towns (up to 20,000 inhabitants)	15	20.27
	Villages	24	32.43
Education	Higher	53	71.62
	Secondary	21	28.38
Employment	Yes	59	79.73
	No	15	20.27
Relationship status	Single	5	6.76
	Informal relationship	7	9.46
	Married	62	83.78
Family type	Two parents	54	72.97
	Single parent	6	8.11
	Family with one biological parent	5	6.76
	Multigenerational family	6	8.11
	Other	3	4.05
Number of children in a household, including partner’s child(ren)	1 child	19	25.68
	2 children	32	43.24
	3 children	16	21.62
	4 children	5	6.76
	5 children	0	0
	6 children	1	1.35
	7 children	1	1.35
Age of the youngest (or only) child in the household	*M* = 7.66, *SD* = 4.97, min. = 0, max. = 22	74	100
Age of the eldest (or only) child in the household	*M* = 11.42, *SD* = 5.50, min. = 0, max. = 24	74	100
Child(ren) with disability	Yes	12	16.22
	No	62	83.78
Time (in hours) spent with child(ren) per day	*M* = 6.57, *SD* = 3.57, min. = 1, max. = 24	74	100

*Note*. As mentioned in Section 2.1, one parent did not indicate their age; therefore, descriptive statistics of age of the total sample were calculated based on a sample of *n* = 73.

**Table 2:** Descriptive statistics for the study variables (*n* = 74).

Variables	McDonald’s Omega (95% CI)	Cronbach’s Alpha (95% CI)	*M*	*SD*	Skewness	Kurtosis	Minimum	Maximum
PACER Behavioral disengagement	0.90 (0.86; 0.94)	0.89 (0.85; 0.93)	22.23	7.66	−0.22	−0.89	5	35
PACER Problem solving	0.86 (0.81; 0.91)	0.86 (0.80; 0.92)	30.66	4.09	−1.20	1.51	16	35
PACER Social support search	0.91 (0.88; 0.94)	0.90 (0.86; 0.94)	30.54	4.49	−1.10	0.66	17	35
PACER Rumination	0.88 (0.83; 0.93)	0.87 (0.79; 0.94)	11.99	6.10	1.91	4.51	5	35
PACER Distraction	0.92 (0.89; 0.95)	0.92 (0.88; 0.95)	25.07	7.04	−0.68	−0.04	8	35
PACER Reappraisal	0.90 (0.86; 0.93)	0.90 (0.82; 0.97)	29.51	4.42	−1.07	2.30	12	35
PACER Acceptance	0.89 (0.85; 0.93)	0.89 (0.81; 0.96)	31.47	3.64	−0.93	0.08	22	35
PACER Expressive suppression	0.91 (0.88; 0.94)	0.91 (0.85; 0.97)	10.72	6.85	1.83	3.26	5	35
PACER Venting	0.83 (0.77; 0.89)	0.83 (0.76; 0.90)	27.68	5.22	−0.79	0.77	11	35
PACER Avoidance	0.92 (0.89; 0.95)	0.92 (0.89; 0.95)	19.04	7.85	−0.04	−0.74	5	35
PBA Parental burnout	0.97 (0.96; 0.98)	0.97 (0.96; 0.98)	30.76	26.32	1.40	1.68	0	118
PACER Adaptive strategies (composite score)	0.93 (0.91; 0.96)	0.93 (0.91; 0.95)	149.86	16.78	−0.95	1.68	86	175
PACER Maladaptive strategies (composite score)	0.94 (0.91; 0.96)	0.93 (0.91; 0.96)	89.04	26.03	0.77	1.47	36	175
PHQ-4 Anxiety	0.61 (0.40; 0.75)	0.61 (0.40; 0.75)	2.51	1.62	0.73	0.05	0	6
PHQ-4 Depression	0.83 (0.73; 0.89)	0.83 (0.72; 0.89)	1.55	1.37	1.03	1.76	0	6
PHQ-4 Total score	0.80 (0.71; 0.86)	0.79 (0.69; 0.85)	4.07	2.69	0.83	0.61	0	12
GBB-8 Somatic complaints	0.88 (0.83; 0.92)	0.88 (0.83; 0.92)	11.93	7.06	0.67	−0.00	0	32
WHO-5 Well-being	0.90 (0.87; 0.94)	0.90 (0.86; 0.93)	12.24	4.84	−0.08	−1.12	4	20
EBQ Negative-Controllability	0.67 (0.55; 0.79)	0.67 (0.54; 0.81)	7.45	3.57	0.93	−0.11	4	16
EBQ Positive-Controllability	0.76 (0.67; 0.85)	0.74 (0.63; 0.85)	7.55	3.71	0.76	−0.61	4	16
EBQ Negative-Usefulness	0.78 (0.69; 0.86)	0.76 (0.66; 0.85)	6.96	3.88	1.68	2.88	4	22
EBQ Positive-Usefulness	0.76 (0.67; 0.85)	0.70 (0.58; 0.81)	5.01	2.34	3.36	12.48	4	16

**Table 3:** Pearson correlations between the study variables (*n* = 74).

Variables	PBA Parental Burnout	PHQ-4 Anxiety	PHQ-4 Depression	PHQ-4 Total Score	GBB-8 Somatic Complaints	WHO-5 Total Score	EBQ Negative-Controllability	EBQ Positive-Controllability	EBQ Negative-Usefulness	EBQ Positive-Usefulness	Number of Significant Correlations (Out of 10)
PACER Behavioral disengagement	0.11	0.05	0.13	0.09	0.05	−0.17	0.13	0.06	0.40 ***	0.23	1
PACER Problem solving	−0.27 *	−0.20	−0.28 *	−0.26 *	−0.16	0.35 **	−0.14	−0.10	−0.03	−0.07	4
PACER Social support search	−0.31 **	−0.22	−0.38 ***	−0.33 **	−0.29 *	0.31 **	−0.19	−0.01	−0.01	0.09	5
PACER Rumination	−0.07	0.04	−0.05	−0.00	−0.19	0.02	0.14	0.10	0.23 *	0.29 *	2
PACER Distraction	0.02	−0.08	0.06	−0.02	−0.00	−0.03	0.05	0.10	0.31 **	0.20	1
PACER Reappraisal	−0.32 **	−0.38 ***	−0.34 **	−0.40 ***	−0.14	0.35 **	−0.25 *	−0.16	−0.12	−0.08	6
PACER Acceptance	0.07	−0.04	−0.01	−0.03	0.16	−0.02	−0.35 **	−0.22	−0.45 ***	−0.36 **	3
PACER Expressive suppression	−0.16	−0.01	−0.08	−0.05	−0.26 *	0.12	0.20	0.15	0.59 ***	0.52 ***	3
PACER Venting	−0.12	−0.24 *	−0.29 *	−0.29 *	−0.06	0.31 **	−0.25 *	−0.26 *	−0.27 *	−0.21	7
PACER Avoidance	0.24 *	0.17	0.26 *	0.23 *	0.10	−0.16	0.10	0.14	0.43 ***	0.20	4
PACER Adaptive strategies (composite score)	−0.25 *	−0.29 *	−0.35 **	−0.35 **	−0.14	0.35 **	−0.31 **	−0.20	−0.22	−0.16	6
PACER Maladaptive strategies (composite score)	0.05	0.05	0.10	0.08	−0.07	−0.07	0.17	0.15	0.54 ***	0.38 ***	2

*Note*. * *p* < 0.05; ** *p* < 0.01; *** *p* < 0.001.

**Table 4:** The results of exploratory factor analysis of ten PACER subscales (*n* = 74).

Variables	Factor 1 “Adaptive Strategies”	Factor 2 “Maladaptive Strategies”
PACER Social support search	0.78	
PACER Reappraisal	0.73	
PACER Problem solving	0.73	
PACER Venting	0.65	
PACER Acceptance	0.62	
PACER Avoidance		0.79
PACER Expressive suppression		0.74
PACER Behavioral disengagement		0.67
PACER Distraction		0.67
PACER Rumination		0.36
Eigenvalues	2.56	2.34
The proportion of total variance for the rotated solution (%)	25.41	23.56

*Note*. For clarity reasons, factor loadings lower than 0.30 are not displayed. Changing rotation from varimax to oblimin does not change the results substantially.

**Table 5:** The results of exploratory factor analysis of ten PACER subscales and four EBQ subscales (*n* = 74).

Variables	Factor 1 “Maladaptive Strategies”	Factor 2 “Adaptive Strategies”	Factor 3 “Emotion Beliefs”
PACER Social support search		0.81	
PACER Reappraisal		0.72	
PACER Problem solving		0.72	
PACER Venting		0.63	
PACER Acceptance		0.59	
PACER Avoidance	0.78		
PACER Expressive suppression	0.73		0.31
PACER Behavioral disengagement	0.68		
PACER Distraction	0.64		
PACER Rumination	0.31		
EBQ Negative-Controllability			0.86
EBQ Positive-Controllability			0.76
EBQ Negative-Usefulness	0.53		0.62
EBQ Positive-Usefulness	0.36		0.52
Eigenvalues	3.67	2.63	1.21
The proportion of total variance for the rotated solution (%)	19.00	18.58	16.08

*Note*. For clarity reasons, factor loadings lower than 0.30 are not displayed. Changing rotation from varimax to oblimin does not change the results substantially.

## Data Availability

The raw data supporting the conclusions of this article will be made available by the authors on request.

## Supplementary Materials

The following supporting information can be downloaded at: https://www.mdpi.com/article/10.3390/children12111428/s1, The Polish version of the Parental Assistance with Child Emotion Regulation (PACER) Questionnaire.

## Abbreviations

The following abbreviations are used in this manuscript:

CI	Confidence interval
EBQ	Emotion Beliefs Questionnaire
ER	Emotion regulation
GBB-8	Giessen Subjective Complaints List-8
KMO	Kaiser–Meyer–Olkin
PACER	Parental Assistance with Child Emotion Regulation
PBA	Parental Burnout Assessment
PHQ-4	Patient Health Questionnaire-4
WHO-5	WHO-Five Well-being Index
