# Supplementary material for: The Polish Version of the Parental Assistance with Child Emotion Regulation (PACER) Questionnaire: Preliminary Psychometric Properties and Links with Parental Burnout, Mental Health Outcomes, and Emotion Beliefs
Source: Children (Basel). 2025 Oct 22;12(11):1428. doi: 10.3390/children12111428 (PMC12651210; doi:10.3390/children12111428)
Supplement: Supplementary file 1 [file children-12-01428-s001.zip › children-3899690-supplementary.pdf]

Supplementary Materials
The Polish version of the Parental Assistance with Child Emotion Regulation (PACER) Questionnaire

Kwestionariusz wsparcia rodzicielskiego w regulacji emocji dziecka

Instrukcja. Poniższe stwierdzenia opisują różne sposoby reagowania na negatywne emocje Twojego dziecka (np. smutek, złość, lęk). Dla każdego stwierdzenia oceń, w jakim stopniu odnosi się ono do Ciebie w większości sytuacji. Proszę zakreśl wybraną odpowiedź dla każdego stwierdzenia.

	Kiedy moje dziecko doświadcza negatywnych emocji…	Zdecydowanie się nie zgadzam	---	---	Ani się zgadzam, ani się nie zgadzam	---	---	Zdecydowanie się zgadzam
1	Pomagam mojemu dziecku wycofać się z aktywności, które mogą wywoływać negatywne emocje.	1	2	3	4	5	6	7
2	Pomagam mojemu dziecku wyjść z każdej sytuacji, która może wywołać w nim negatywne emocje.	1	2	3	4	5	6	7
3	Pomagam mojemu dziecku wyjść z sytuacji, która może wywołać negatywne emocje, a następnie pomagam mu zaangażować się w inną aktywność.	1	2	3	4	5	6	7
4	Pomagam mojemu dziecku przestać robić wszystko, co wywołuje w nim negatywne emocje.	1	2	3	4	5	6	7
5	Wycofuję moje dziecko z danej sytuacji, kiedy wywołuje ona w nim negatywne emocje.	1	2	3	4	5	6	7
6	Pomagam mojemu dziecku dokładnie przemyśleć różne rozwiązania jego problemów.	1	2	3	4	5	6	7
7	Pomagam mojemu dziecku rozwiązywać problemy, które wywołują w nim negatywne emocje.	1	2	3	4	5	6	7
8	Pomagam mojemu dziecku rozważać różne sposoby rozwiązywania problemów.	1	2	3	4	5	6	7
9	Wspieram moje dziecko w poszukiwaniu rozwiązań jego problemów.	1	2	3	4	5	6	7
10	Pomagam mojemu dziecku podjąć działania, żeby rozwiązać problem.	1	2	3	4	5	6	7
11	Pomagam mojemu dziecku znaleźć osoby, które mogą udzielić mu pomocy (w tym mnie).	1	2	3	4	5	6	7
12	Pomagam mojemu dziecku dotrzeć do osób, z którymi może nawiązać kontakt (w tym do mnie).	1	2	3	4	5	6	7
13	Wspieram moje dziecko w dotarciu do krewnych i przyjaciół, którzy mogą udzielić mu wsparcia (w tym do mnie).	1	2	3	4	5	6	7
14	Pomagam mojemu dziecku odnaleźć ludzi, którzy mogą być dla niego fizycznie dostępni (w tym mnie).	1	2	3	4	5	6	7
15	Zachęcam moje dziecko do nawiązywania kontaktów z innymi (w tym ze mną).	1	2	3	4	5	6	7

	Kiedy moje dziecko doświadcza negatywnych emocji…	Zdecydowanie się nie zgadzam	---	---	Ani się zgadzam, ani się nie zgadzam	---	---	Zdecydowanie się zgadzam
16	Pomagam mojemu dziecku odtworzyć w myślach to, co wywołuje w nim negatywne emocje.	1	2	3	4	5	6	7
17	Pomagam mojemu dziecku ciągle myśleć o tym, co wywołuje w nim negatywne emocje.	1	2	3	4	5	6	7
18	Zachęcam moje dziecko, aby nieustannie zastanawiało się, dlaczego przeżywa negatywne emocje.	1	2	3	4	5	6	7
19	Pomagam mojemu dziecku ciągle odtwarzać w myślach emocjonalnie negatywne przeżycia.	1	2	3	4	5	6	7
20	Pomagam mojemu dziecku nieustannie myśleć o sytuacjach, które są dla niego denerwujące lub wywołują negatywne emocje.	1	2	3	4	5	6	7
21	Pomagam mojemu dziecku znaleźć sposoby na odwrócenie uwagi od negatywnych emocji.	1	2	3	4	5	6	7
22	Pomagam mojemu dziecku odwrócić uwagę od negatywnych emocji poprzez znalezienie innej aktywności.	1	2	3	4	5	6	7
23	Pomagam mojemu dziecku oderwać myśli od tego, co wywołuje w nim negatywne emocje.	1	2	3	4	5	6	7
24	Pomagam mojemu dziecku odwrócić uwagę od tego, co wywołuje w nim negatywne emocje.	1	2	3	4	5	6	7
25	Pomagam mojemu dziecku myśleć o czymś innym niż o tym, co wywołuje w nim negatywne emocje.	1	2	3	4	5	6	7
26	Pomagam mojemu dziecku spojrzeć na daną sytuację w pozytywnym świetle.	1	2	3	4	5	6	7
27	Pomagam mojemu dziecku spojrzeć na daną sytuację z innej perspektywy.	1	2	3	4	5	6	7
28	Pomagam mojemu dziecku dostrzec pozytywne aspekty w sytuacji, która wywołuje w nim negatywne emocje.	1	2	3	4	5	6	7
29	Pomagam mojemu dziecku radzić sobie z emocjami poprzez zmianę jego sposobu myślenia o danej sytuacji.	1	2	3	4	5	6	7
30	Zachęcam moje dziecko do dostrzegania pozytywnych aspektów jego negatywnych emocji.	1	2	3	4	5	6	7
31	Pomagam mojemu dziecku zrozumieć, że odczuwanie negatywnych emocji jest naturalne.	1	2	3	4	5	6	7
32	Pomagam mojemu dziecku zaakceptować jego negatywne emocje.	1	2	3	4	5	6	7
33	Pomagam mojemu dziecku zaakceptować to, jak się czuje, kiedy nie może zmienić danej sytuacji.	1	2	3	4	5	6	7
34	Mówię mojemu dziecku, że negatywne emocje są naturalne.	1	2	3	4	5	6	7
35	Tłumaczę mojemu dziecku, że w pewnych sytuacjach warto zaakceptować negatywne emocje.	1	2	3	4	5	6	7

	Kiedy moje dziecko doświadcza negatywnych emocji…	Zdecydowanie się nie zgadzam	---	---	Ani się zgadzam, ani się nie zgadzam	---	---	Zdecydowanie się zgadzam
36	Pomagam mojemu dziecku nie wyrażać/okazywać negatywnych emocji.	1	2	3	4	5	6	7
37	Pomagam mojemu dziecku ukrywać jego emocje przed innymi.	1	2	3	4	5	6	7
38	Pomagam mojemu dziecku ukrywać objawy negatywnych emocji (np. płacz, krzyk).	1	2	3	4	5	6	7
39	Pomagam mojemu dziecku ukrywać negatywne emocje przed innymi w taki sposób, aby nie byli w stanie rozpoznać u niego tych emocji.	1	2	3	4	5	6	7
40	Zachęcam moje dziecko do ukrywania jego negatywnych emocji przed innymi.	1	2	3	4	5	6	7
41	Pomagam mojemu dziecku rozmawiać otwarcie z innymi.	1	2	3	4	5	6	7
42	Pomagam mojemu dziecku rozmawiać o sytuacji lub problemie, przez który tak się czuje.	1	2	3	4	5	6	7
43	Zachęcam moje dziecko, aby częściej rozmawiało z innymi o tym, co czuje.	1	2	3	4	5	6	7
44	Zachęcam moje dziecko, by zwierzało się innym ze swoich trudności.	1	2	3	4	5	6	7
45	Pomagam mojemu dziecku wyrażać/okazywać negatywne emocje przed innymi.	1	2	3	4	5	6	7
	**Zanim moje dziecko doświadczy negatywnych emocji…**	**Zdecydowanie się nie zgadzam**	**---**	**---**	**Ani się zgadzam, ani się nie zgadzam**	**---**	**---**	**Zdecydowanie się zgadzam**
46	Kiedy tylko to możliwe, pomagam mojemu dziecku unikać potencjalnie nieprzyjemnych sytuacji.	1	2	3	4	5	6	7
47	Pomagam mojemu dziecku trzymać się z dala od sytuacji, które mogą wywołać u niego negatywne emocje.	1	2	3	4	5	6	7
48	Podejmuję działania chroniące moje dziecko przed sytuacjami, które mogą wywołać u niego negatywne emocje.	1	2	3	4	5	6	7
49	Skłaniam moje dziecko do trzymania się z dala od sytuacji, które mogą wywołać w nim negatywne emocje.	1	2	3	4	5	6	7
50	Pomagam mojemu dziecku unikać robienia rzeczy, które mogą wywołać u niego negatywne emocje.	1	2	3	4	5	6	7

Obliczanie wyników PACER

Kwestionariusz wsparcia rodzicielskiego w regulacji emocji dziecka poddaje ocenie stosowanie 10 strategii regulacji emocji, których używają rodzice w celu regulacji emocji dziecka. PACER składa się z 50 pytań, po 5 na każdą z 10 strategii regulacji emocji. Poniższa tabela 1 obrazuje wszystkie podskale, a także wskazuje, jak je obliczyć.

Tabela 1
Obliczanie wyników PACER

	Podskale	Obliczanie wyników
1	Wycofywanie się	Suma wyników twierdzeń 1–5
2	Rozwiązywanie problemów	Suma wyników twierdzeń 6–10
3	Poszukiwanie wsparcia społecznego	Suma wyników twierdzeń 11–15
4	Ruminacja	Suma wyników twierdzeń 16–20
5	Odwracanie uwagi	Suma wyników twierdzeń 21–25
6	Przeformułowanie poznawcze	Suma wyników twierdzeń 26–30
7	Akceptacja	Suma wyników twierdzeń 31–35
8	Tłumienie ekspresji	Suma wyników twierdzeń 36–40
9	Wyrażanie emocji	Suma wyników twierdzeń 41–45
10	Unikanie	Suma wyników twierdzeń 45–50
